# Postpartum ovarian vein thrombosis after cesarean delivery: a case report

**DOI:** 10.1186/1752-1947-2-105

**Published:** 2008-04-09

**Authors:** Pedro Royo, Alberto Alonso-Burgos, Manuel García-Manero, Ramón Lecumberri, Juan Luis Alcázar

**Affiliations:** 1 Obstetrics and Gynecology Department, Clínica Universitaria de Navarra, Avda Pío XII, 31008 Pamplona, Spain; 2 Radiology Department, Clínica Universitaria de Navarra, Avda Pío XII, 31008 Pamplona, Spain; 3 Hematology Department, Clínica Universitaria de Navarra, Avda Pío XII, 31008 Pamplona, Spain

## Abstract

**Introduction:**

Postpartum ovarian vein thrombosis is an uncommon complication; incidence varies between 0.002% and 0.05%. It most often occurs during the 2–15 days following delivery.

**Case presentation:**

A 22-year-old pregnant woman at term presented to hospital with uterine contractions, abdominal pain, nausea and vomiting. After delivery an ovarian vein thrombosis was diagnosed.

**Conclusion:**

Low-molecular weight heparin with broad-spectrum antibiotics are the accepted therapy in non-complicated cases of postpartum ovarian vein thrombosis.

## Introduction

In this report we describe a case of postpartum ovarian vein thrombosis (POVT), a rare complication of pregnancy and delivery that increases maternal morbidity. The risk factors, physiopathology features, diagnostic approach and therapeutic options are described.

Ovarian vein thrombosis is an uncommon complication. Computed tomography (CT) is most useful in making the diagnosis. Heparin and antibiotics are the accepted therapy in non-complicated cases.

## Case presentation

A 22-year-old woman who was pregnant at term presented to our hospital with uterine contractions, abdominal pain, nausea and vomiting. The hemogram, ionogram, coagulation work-up and urine culture were normal. There was no relevant family history of disease. Past medical history included one abortion three years previously, use of oral contraceptives for several years, no history of deep vein thrombosis (DVT) and no history of hypertension. In the present pregnancy, there had been a first trimester threat of miscarriage. She was immunized for rubella. There were negative serologies for hepatitis B virus (HBV), varicella zoster virus (VZV), human immunodeficiency virus (HIV) and toxoplasma. Rectal and vaginal cultures were negative for hemolytic streptococci.

After admission, a non-stressant test was performed. Fetal tachycardia (170 bpm) with a non-reactive pattern was detected. A fetal Doppler sonography revealed a 'brain-sparing' effect with a cerebroplacental ratio of 0.75 (normal > 1) [1]. An urgent cesarean delivery was performed. Neonatal weight at birth was 2,970 g (P50), the Apgar score was 9–10 and fetal gasometry values were normal.

During surgery, a large and bilateral varicose uterine plexus was observed. DVT prophylaxis was administrated. Bemiparin (Hibor^®^) 3500 UI sc/24 hours (first administration eight hours after a cesarean delivery) and elastic compression stockings were used for this purpose during admission. Eight hours after the patient was discharged, she returned with abdominal pain, fever (38.3°C) and dyspnea. A review of the Pfannenstiel incision showed it was in good condition with no sign of infection. Abdominal examination revealed intense tenderness in the left iliac fossa. Vaginal examination showed odorless loquia and pain with uterine mobilization. A blood test with white blood cell count revealed leukocytosis (9,400) with neutrophilia (83%). Urine culture values were normal.

**Figure 1:**
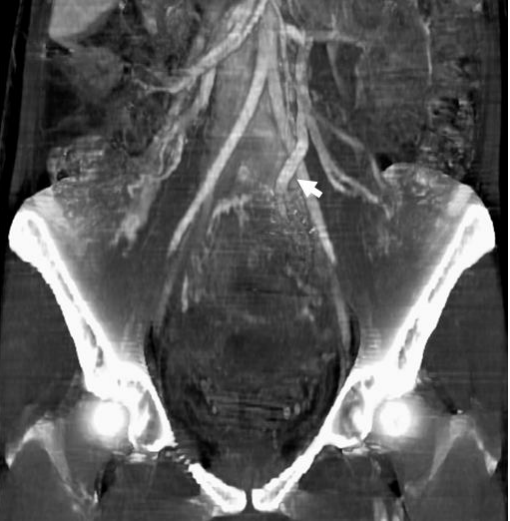
**Contrast-enhanced CT-scan images with maximum intensity projections showing an increased diameter of the left ovarian vein**. Three-dimensional reconstruction and anteroposterior view in which an increased diameter of the left ovarian vein (arrow) can be observed as an indirect sign of thrombosis.

**Figure 2:**
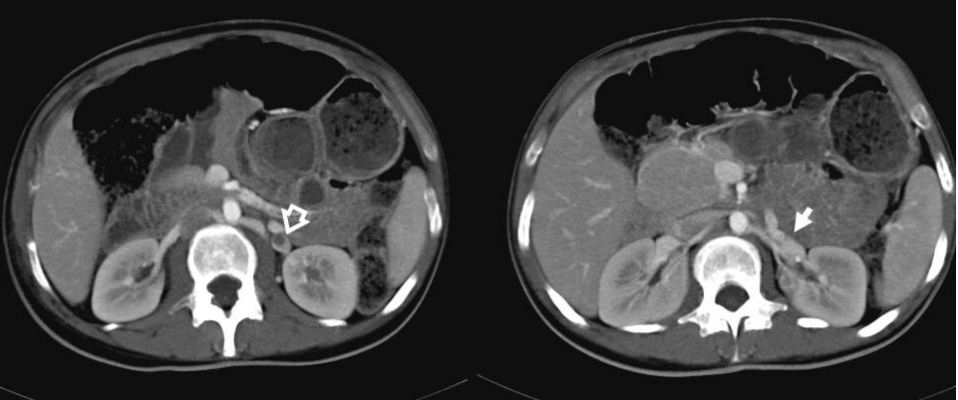
**Contrast-enhanced CT-scan images with maximum intensity projections showing an intravascular filling defect in the left ovarian vein**. 3D-reconstruction showing an intravascular filling defect in the left ovarian vein (open arrow) related to thrombosis. No thrombus progression into the left renal vein was observed (arrow).

**Figure 3:**
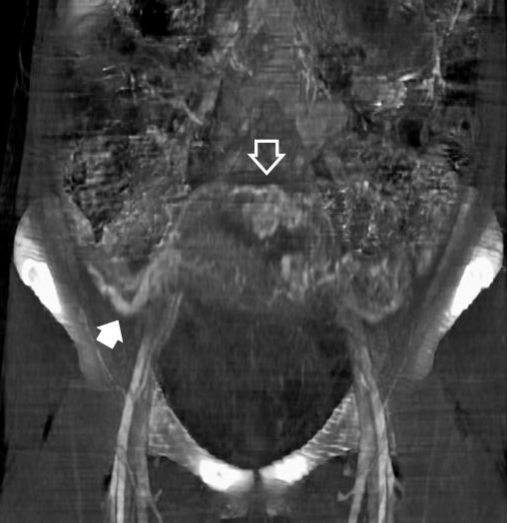
**Contrast-enhanced CT-scan images with maximum intensity projections showing a large number of uterine variceal vessels**. Three-dimensional reconstruction and anteroposterior view in which a large number of uterine variceal vessels can be seen (open arrow) as well as collateral venous drainage pathways (solid arrow).

A small rectus abdomini hematoma was revealed on abdominal and transvaginal ultrasound scans. A CT scan was requested to assess the late-postoperative abdominal pain. After intravenous contrast injection, a complete left POVT involving the junction of the ovarian vein with the renal vein was demonstrated. (Figures 1, 2, 3).

Cultures of the haematoma showed growth of *Staphylococcus aureus*. Bemiparin was increased up to 5,000 UI sc/24 hours and amoxicilin-clavulanic acid (Augmentine^®^) was started (1 g ev/8 hours). The specific coagulation work-up showed an S protein deficiency of 29% (normal range 70–120%) but normal values for homocysteine, antithrombin III, C protein, antiphospholipid antibodies, Leyden V factor and prothrombin gene G20210A mutation.

Seven days later, the patient was discharged. Amoxicilin-clavulanic acid was continued over the next four days and bemiparin (5,000 UI sc/24 hours) was continued for the following four months, with a decreased dose (3,500 UI sc/24 hours) for the next two months and then the treatment was ceased.

## Discussion

POVT incidence varies between 0.002% and 0.05% (see [2-4]) and DVT incidence is many times more frequent during pregnancy [3]. Cesarean delivery increases the risk of thrombosis to 1–2% (see [2,3]) and multiparity has also been identified as a risk factor for thrombosis [5]. Thrombophilias are present in 50% of POVT patients [3]. All of these features explain why pregnancy is a well-known 'hypercoagulable state'. The uterus increases in size and its blood flow also increases. These changes may impede venous outflow from the lower limbs [6] generating pelvic vein stasis, increased levels of I, II, VII, IX and X coagulation factors [5], increased thrombin generation, fibrinolysis inhibition for up to 72 hours after delivery, increased platelet adhesion and decreased C and S anticoagulant proteins, which may be acquired or hereditary [2]. These proteins inactivate factors Va and VIIIa and also the inhibitor of the plasminogen activator, increasing the risk of thrombosis during pregnancy by 7–17%. [7].

These gestational prothrombotic changes can complicate the real diagnosis of thrombophilias in pregnancy [7].

Here we have reported an uncommon case of left POVT. The right vein is involved in 70–90% of cases and bilateral thrombosis is present in 11–14% of cases [4,8]. Many hypotheses have tried to explain why the right vein is implicated in a larger number of cases of POVT. The main theory reported is that the right vein is longer than the left vein. Therefore, the right vein may be more likely to be compressed during the dextrorotation of the pregnant uterus. In addition, the characteristic retrograde and slow flow in the right vein during the postpartum stage may also increase the risk of right thrombosis [2-7].

The classic presentation of POVT includes pelvic and/or flank pain and fever during the 15 days after delivery, leukocytosis and a homolateral palpable mass [4,7]. Some of these symptoms were present in our case.

The differential diagnosis of POVT includes acute appendicitis, intestinal volvulus, broad-ligament hematoma, adnexal torsion, abscess, pyelonephritis, retroperitoneal lymphadenopathy and puerperal endometritis [2-5]. Puerperal endometritis has been postulated as a possible cause of POVT. Anaerobic bacteria, which are usually present in the lower genital tract, with or without endometritis, are able to generate an endothelial injury and, stasis with secondary thrombosis of the pelvic veins. The bacteria might reach the ovarian veins from the septic endometrium by crossing the uterine and vaginal venous and lymphatic plexus [4,5].

A correct diagnosis of POVT can be made by ultrasonography, magnetic resonance imaging (MRI) or CT scan with a sensitivity of 52%, 92% and 100%, respectively [3]. Ultrasound has previously been widely used for the evaluation of POVT [9]. The Doppler ultrasound can also be used for the diagnosis and later follow-up of flow restoration [10]. Magnetic resonance (MR) angiography can provide a better and more reliable visualization of the vascular systems and the coronal source images are useful in evaluating the extent of a thrombus [11]. A helical CT-angiography study with bolus injection of iodinated contrast material and conventional venography provides an accurate method for diagnosing POVT and this is considered the standard method for diagnosis of this condition. Dilated, thick-walled ovarian veins with rim enhancement and a central hypodensity are considered to be the main CT imaging findings of POVT [12,13] (Figure 2). The severity of this disease is related to the extension of the thrombosis into the inferior cava vein and the hazard of pulmonary embolism which occurs in 13% of cases with a 4% mortality [2]. These findings must be confirmed or excluded using either MR angiography or CT pulmonary-angiography at the time of diagnosis of POVT. Recent advances in helical CT and the development of multiplanar reconstructions and maximum intensity projections (MIP) have allowed a global and immediate approach to a large number of pathologies of the vascular system, including POVT.

Most studies suggest the use of low molecular weight heparin (LMWH) and broad-spectrum antibiotics in non-complicated cases of POVT, but there is no consensus about the type, dose or duration of treatment [5]. LMWH prophylaxis prevented 48% of symptomatic pulmonary embolisms, 48% of symptomatic DVTs and 51% of asymptomatic DVTs in acutely ill medical inpatients [11].

Fibrinolytic drugs have not shown enough efficacy to be recommended in the management of POVT. When medical support does not control the symptoms or a high risk of pulmonary embolism is present, then endovascular or surgical procedures, such as thrombectomy, cava filters, ovarian or cava vein ligature, may be indicated [2,4-7].

## Conclusion

Postpartum ovarian vein thrombosis is an uncommon complication of the postpartum period. Thrombophilias and puerperal endometritis are the most likely causes of this type of thrombosis. Helical CT-angiography is the investigation of choice in diagnosis. LMWH with broad-spectrum antibiotics is effective as the initial treatment in cases without pulmonary embolism or wide involvement of the thrombus in the inferior cava vein.

## Competing interests

The author(s) declare that they have no competing interests.

## Authors' contributions

PR reviewed the literature and wrote the case description and discussion. AAB was responsible for the CT imaging files and literature comments on radiology.

MGM, as a specialist in obstetrics and gynecology, revised and corrected all areas in the text covering this field. RL, as a specialist in hematology, revised and corrected all areas in the text covering this field. JLA, as a specialist in obstetric and gynecology imaging, revised and corrected all relevant areas of the text.

## Consent

Written informed consent was obtained from the patient for publication of this case report and accompanying images. A copy of the written consent is available for review by the Editor-in-Chief of this journal.
